# Editorial: Global excellence in food chemistry

**DOI:** 10.3389/fnut.2022.1039724

**Published:** 2022-10-17

**Authors:** Alaa El-Din A. Bekhit, Jesus Simal-Gandara, A. M. Abd El-Aty

**Affiliations:** ^1^Department of Food Science, University of Otago, Dunedin, New Zealand; ^2^Nutrition and Bromatology Group, Analytical and Food Chemistry Department, Faculty of Food Science and Technology, University of Vigo, Ourense, Spain; ^3^Department of Pharmacology, Faculty of Veterinary Medicine, Cairo University, Giza, Egypt; ^4^Department of Medical Pharmacology, Medical Faculty, Ataturk University, Erzurum, Turkey

**Keywords:** food safety, bioactives, antimicrobial, protein, rapid detection

The current global changes in economic, social, and technological production systems of food necessitate developing innovative solutions and strategies that ensure maximum utilization of food resources to produce desirable and wholesome food products. Food chemistry and related research activities are arguably the core of research activities that ensure the achievement of the above goals. This Research Topic is aimed at capturing prominent food chemistry research activities to provide recent insights and current research activities to meet the above goals. This Research Topic provides a balanced collection of original research, reviews, and new methods contributions, authored by experts in the field. The studies reported in the present Research Topic can be generally categorized into the following themes: food safety research that was concerned with the detection and quantification of pesticides and antimicrobial agents; studies on bioactive compounds, their stability and biofunctionalities; fractionation of pea protein, and others.

## Food safety

Given the obvious importance of food safety, this theme area has been a major topic of interest in this Research Topic. The use of pesticides to protect crops is crucial for financial and food security reasons. However, pesticide residue in agricultural produce is known to have harmful effects on human health, especially those that are consumed fresh without any effective pre-processing treatments (e.g., peeling, sanding, other surface treatment, or thermal treatments). Considering these aspects, Park et al. investigated common food preparations (washing and blanching) on residual cyantraniliprole contents in spinach using ultrahigh-performance liquid chromatography-tandem mass spectrometry (UHPLC–MS/MS). A synergistic effect for washing with a neutral detergent and blanching for 5 min in boiling water led to a 93.5% reduction in cyantraniliprole content. Furthermore, Zhai R. et al. investigated the content of imidacloprid in Chinese chives using a quick, easy, cheap, effective, rugged, and safe (QuEChERS) method combined with liquid chromatography-tandem mass spectrometry (LC–MS/MS). The authors reported good linearity (*R*^2^ = 0.9988), a limit of quantification of ≤ 8.07 × 10^4^ mg/kg, and a recovery range of 78.34–91.17%. The dissipation dynamics of imidacloprid in Chinese chives followed first-order kinetics, and the pesticide had a half-life of 2.92 days. Applying the method to commercial samples revealed that the imidacloprid contents in Chinese chives (0.00923–0.166 mg/kg) were below the maximum residue limit (MRL) of 1 mg/kg. Similar research that investigated dissipation and contents of fenpyroximate acaricide in/on guava, orange, and eggplant under open field conditions is reported by Malhat et al. The authors reported the kinetics of the pesticide in all crops to follow a first-order kinetics model with half-lives of 1.7, 2.2, and 1.9 days for eggplants, guavas, and oranges, respectively. An important finding of the study was that 3 and 7 days of the postharvest interval were proposed for oranges and eggplant to reach a level that complies with MRLs in these agricultural products. For guava, no postharvest interval time was proposed due to the absence of MRL.

Concerns over the translocation of phthalates from food packaging to beef were investigated by Baranenko et al. The contents of dimethyl terephthalate (DMTP), di-n-butyl phthalate (DnBP), and diisooctyl phthalate (DiOP) were determined in commercial beef samples. The authors found that mince beef had a higher phthalate content than sliced beef due to the larger contact area and the presence of distributed fat on the surface of the minced meat in direct contact with the packaging material.

Interest in rapid methods for the detection and quantification of pesticides is also reported in this Research Topic. A colloidal gold immunochromatographic strip was developed for the rapid detection of aldicarb in vegetables (leeks and cabbages) (Shen et al.). The strips were accurately capable of identifying positive samples but detected false positives for negative samples.

A fast method for simultaneous quantification of chloramphenicol, thiamphenicol, florfenicol, and florfenicol amine in meat and seafood was reported by Jung et al. The authors employed a QuEChERS extraction method coupled with a liquid chromatography-tandem mass spectrometry (LC–MS/MS) method that offers several advantages. The developed method had limits of detection of 0.005–3.1 μg/kg and limits of quantification of 0.02–10.4 μg/kg. The method was verified using various animal-derived products. The authors concluded that the developed method was versatile, sensitive, and suitable for the quantification of antimicrobials in animal products.

A competitive assay combining aptamer (DNA)-specific recognition and bimetallic nanozyme gold@platinum (Au@Pt) catalysis was developed by Chen et al. to quantify carbendazim, a fungicide used in agricultural products. The developed method had a limit of detection of 0.038 ng/mg, and a good correlation was established between the developed method and parallel analysis carried out using liquid chromatography coupled with mass spectrometry analysis. The authors concluded that the competitive assay based on dual-mode Au@Pt-DNA biosensors has a high potential for detecting fungicides in agroproducts. A similar approach was used by Zhang et al. to determine fenvalerate contents in vegetables using the immunocompetition method that employed an electrospun fiber membrane containing a fenvalerate hapten-mouse IgG-Eu fluorescent probe.

## Bioactives

The effect of terpinen-4-ol (the major component in tea tree oil) on inflammatory bowel disease (IBD) was evaluated using a cell model [lipopolysaccharide (LPS)-induced intestinal epithelial cell barrier function impairment in intestinal porcine epithelial cell lines] and an animal model [dextran sulfate sodium (DSS)-induced IBD in mice] (Yong et al.). Terpinen-4-ol was successful in protecting intestinal porcine epithelial cell lines against LPS-induced damage and attenuated DSS-induced colitis in mice. The authors demonstrated that terpinen-4-ol activity was due to the promotion of tight junction (TJ) proteins. Additionally, Zhai X. et al. investigated the use of ultrasound-assisted extraction techniques to accelerate the extraction of cold coffee brewing. The authors compared the physicochemical characteristics and non-volatile and volatile compounds of ultrasound-extracted coffee extracts with hot brewing and conventional static cold brewing methods. The ultrasound technique resulted in a faster extraction time (1 h compared to 12 h in the traditional cold extraction process) and higher total dissolved solids (6–26%), total lipids (10–21%), proteins (26–31%), and titrated acids (12–15%) than the static cold brews. A “pour-over” extraction with hot water (92°C, 3 min, 3 stages) resulted in higher caffeine, chlorogenic acid, and trigonelline contents compared to boiling (95°C, 5 min), cold brewing for 12 h, and ultrasound-assisted extraction for 1 h. The volatile profiles of the ultrasound-assisted cold brew extracts were similar to static cold ones, but both of these samples were different from the hot brewed samples. Overall, the research showed the usefulness of ultrasound in enhancing the extraction process without compromising the coffee flavor.

A Q Exactive Hybrid Quadrupole-Orbitrap Mass Spectrometer was used for the identification of flaxseed cyclolinopeptides, cyclic peptides that have been reported to have a wide range of bioactivities, such as inhibition of osteoclast differentiation and antimalarial, immunosuppressive, and antitumor activities (Fojnica et al.). The study identified twelve cyclolinopeptides, and the stability and degradation of the cyclolinopeptides in flaxseed oil were studied over 60 days at room temperature and 90°C. The majority of the peptides were rapidly degraded at 90°C and slowly degraded at room temperature with significant degradation profiles and rates among the various peptides.

Microencapsulation of plant (red beet, broccoli, and spinach leaf) phenolic extracts was carried out using a complex coacervation method to improve their stability (Soliman et al.). The microcapsules were tested for their physicochemical properties, sensory attributes, and bioactivities in a rat model. Overall, these microcapsules were found to support brain health, improve metabolic strategies and neurobehavioral systems and enhance protein biosynthesis in AlCl3 (100 mg/kg body weight/d)-treated rats. A similar study investigated the protective effects of D-cycloserine and L-serine in the AlCl3-induced experimental rat model of Alzheimer's disease and reported positive results (Tozlu et al.).

Bioactives and their health benefits have received comprehensive reviews that summarize up-to-date information on bioactive peptides from food and byproducts (Zaky et al.; Mitra et al.), polysaccharides in *Cordyceps militaris* (Miao et al.), and protein-phenolic interactions (Yilmaz et al.).

## Fractionation of pea protein

Asen and Aluko investigated the aggregation of pea proteins as affected by pH (3.0, 5.0, 7.0, or 9.0), heat treatment (100°C for 30 min), and fractionation (<30, 30–50, and >50 kDa fractions). The >50 kDa fractions had higher protein content than the other fractions. Within this fraction, different protein aggregation temperatures were found (124.30, 190.66, 206.33, and 203.17°C for pH 3.0, 5.0, 7.0, and 9.0, respectively), which were higher than that of the pea protein concentrate (74.45°C). The >50 kDa fractions obtained at pH 3.0, 7.0, and 9.0 had better solubility, oil holding capacity, protein content, foam capacity, foam stability, water holding capacity, and surface hydrophobicity. Overall, the study demonstrated the ability to manipulate the techno-functionality of the pea protein isolate through the manipulation of pH and fraction.

### Miscellaneous

A meta-analysis for autoclaving-cooling conditions that result in an increased resistant starch content in starchy foods was reported by Faridah et al., and technologies used for rice wine production were reviewed by Pei et al.

## Author contributions

All authors listed have made a substantial, direct, and intellectual contribution to the work and approved it for publication.

## Conflict of interest

The authors declare that the research was conducted in the absence of any commercial or financial relationships that could be construed as a potential conflict of interest.

## Publisher's note

All claims expressed in this article are solely those of the authors and do not necessarily represent those of their affiliated organizations, or those of the publisher, the editors and the reviewers. Any product that may be evaluated in this article, or claim that may be made by its manufacturer, is not guaranteed or endorsed by the publisher.

